# Chronic Hyperuricemia, Uric Acid Deposit and Cardiovascular Risk

**DOI:** 10.2174/1381612811319130011

**Published:** 2013-04

**Authors:** Davide Grassi, Livia Ferri, Giovambattista Desideri, Paolo Di Giosia, Paola Cheli, Rita Del Pinto, Giuliana Properzi, Claudio Ferri

**Affiliations:** University of L’Aquila - Department of Internal Medicine and Public Health - San Salvatore Hospital - Italy; # University “Sapienza - II Faculty of Medicine - Rome - Italy

**Keywords:** Uric acid, allopurinol, febuxostat, metabolic syndrome, cardiovascular risk.

## Abstract

Hyperuricemia is commonly associated with traditional risk factors such as dysglicemia, dyslipidemia, central obesity and abnormal blood pressure, *i.e*. the metabolic syndrome. Concordantly, recent studies have revived the controversy over the role of circulating uric acid, hyperuricemia, and gout as an independent prognostic factor for cardiovascular morbidity and mortality. In this regard, different studies also evaluated the possible role of xanthine inhibitors in inducing blood pressure reduction, increment in flow-mediated dilation, and improved cardiovascular prognosis in various patient settings. The vast majority of these studies have been conducted with either allopurinol or its active metabolite oxypurinol, i.e. two purine-like non-selective inhibitors of xanthine oxidase. More recently, the role of uric acid as a risk factor for cardiovascular disease and the possible protective role exerted by reduction of hyperuricemia to normal level have been evaluated by the use of febuxostat, a selective, non purine-like xanthine oxidase inhibitor. In this review, we will report current evidence on hyperuricemia in cardiovascular disease. The value of uric acid as a biomarker and as a potential therapeutic target for tailored old and novel “cardiometabolic” treatments will be also discussed.

## INTRODUCTION

Uric acid is the final product of purine nucleotide catabolism. In particular, purine nucleotides are derived from both endogenous (*de novo *molecule synthesis and nucleic acid breakdown) and exogenous sources (alimentary intake) [1-3]. *De novo* synthesis of purines depends on the compound 5-phosphoribosyl-l-pyrophosphate, which is converted enzymatically to inosinic acid. In turn, it may additionally either be converted into bases for inclusion into nucleic acids or be broken down into xanthine to form uric acid [1-3]. Purine nucleotide synthesis can also occur through the activities of two different enzymes, catalysing the single-step synthesis of a purine nucleotide from a purine base substrate. During the reverse process, the intermediate breakdown product hypoxanthine can be ‘salvaged’ by the enzyme hypoxanthine-guanine phosphoribosyl transferase and then re-incorporated into nucleic acid. The whole pathway is tightly regulated and controlled by feedback inhibition [1-3]. 

Uric acid is a weak acid, with a ionization constant of acid (pKa) of 5.75-10.3. At the physiological pH of 7.40 of the extracellular compartment, 99% of uric acid is in the ionized form as urate (as monosodium urate in blood and as potassium, ammonium and calcium urate in urine). In the urinary tract, where pH can fall to 5.7, acid uric formation is favored. 

Deoxyribonucleotides and purine nucleotides catabolism leads to uric acid production. Hypoxanthine and xanthine are the intermediate products of this catabolism. Xanthine oxidase catalyzes the oxidation of xanthine to uric acid. Uric acid is the final oxidation product of purine catabolism, which means that it cannot be further metabolized. The kidneys excrete two-thirds of the total uric acid amount that is produced daily, while the remaining one-third is broken down by intestinal flora and excreted in the stool [3, 4]. 

Physiological daily amount of endogenous and exogenous uric acid is about 700 mg, which is balanced by an equal output via feces and urine. As previously stated, 30% of uric acid is broken down by intestinal flora and expelled through the stool, while the remaining 70% (approximately 500 mg per day) is excreted unchanged through the kidneys [1-4].

As a consequence, under normal circumstances uric acid production and excretion are carefully balanced processes [3, 4]. 

In most mammals, the enzyme *uricase* (urate oxidase) oxidizes uric acid to allantoin. Allantoin is highly soluble in water, therefore it does not accumulate in crystals and it is excreted unchanged through urine. Consequently, this makes urate oxidase very effective in lowering uric acid levels [5-7]. Unfortunately, urate oxidase is not a functional human enzyme, probably due to mutations occurring during the Myocene, and uric acid water-solubility is limited. As a result, humans but not other mammals can develop hyperuricemia and uric acid crystals can accumulate in human tissues and in the urinary tract, causing chronic hyperuricemia-related disease.

Despite the modest antioxidant activity exerted by uric acid [8], hyperuricemia is a potentially harmful condition. It favors precipitation of uric acid crystals in joints and tissues, leading to complications such as gout, nephrolithiasis and chronic nephropathy.

Besides these well-known conditions, hyperuricemia has been also recently associated with hypertension, metabolic syndrome and cardiovascular diseases, resulting in an effective therapeutic target for cardiovascular prevention [9].

## HYPERURICEMIA AND GOUT 

Hyperuricemia is defined as a plasma uric acid level greater than 6.8 mg/dL at physiological temperature (37°C) and neutral pH [1, 2, 6]. Nevertheless, recent evidence has pointed out the importance of adapting this range to the population examined. For example, only serum uric acid levels lower than 6.0 mg/dL have to be considered normal in cronic hyperuricemic and gouty patients. As a matter of fact, this is the sole serum uric acid level truly preventing uric acid crystal deposition.

Hyperuricemia is a very common condition, being usually caused by an unhealthy lifestyle that is mainly represented by a poor diet exceeding in purine nucleotides, protein, alcohol, and carbohydrates intake [1-6]. In addition, various drugs must be considered potentially dangerous for purine nucleotides metabolism in patients having cardiovascular comorbidities and risk factors. Thiazides and loop diuretics both frequently cause hyperuricemia [6]. Similarly, low-dose aspirin (primary and secondary cardiovascular prevention) decreases kidney excretion and thereby increases uric acid blood levels [2, 4].

**Fig. (1):**
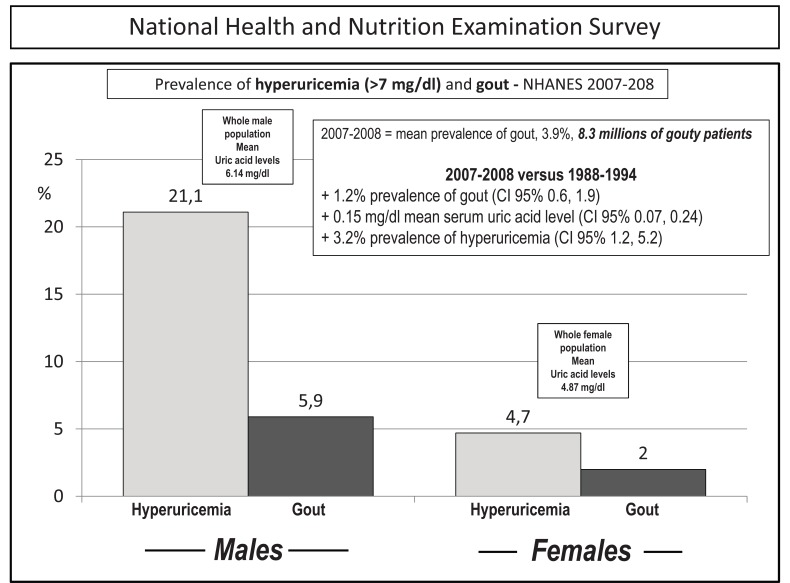
Prevalence of hyperuricemia and gout in the US population in the last two available National Health and Nutrition Examination Surveys. Of note, mean uric acid levels were in the whole male and female populations = 6.14 mg/dl and 4.87 mg/dL, respectively, thus implying marked increments in both hyperuricemia and gout during the last decade (inset). Modified from ref 13.

As already stated, chronic hyperuricemia may cause uric acid precipitation in joints and tissues. Urate deposit may be asymptomatic, and further deposition may continue silently until clinical manifestations such as erosive and deforming arthritis, nephrolitiasis and chronic nephropathy occur. This pathological evolution depends on numerous factors, both endogenous and exogenous, but persistently high levels of serum uric acid, *i.e. *> 6.0 mg/dL, are indeed the most important ones. Depending on the cut off value, a prevalence of up to 15-20% has been reported for hyperuricemia in population-based studies, with urate deposition in about 4% of the cases (Fig. **1**) [10-13]. This makes urate deposition the most common type of arthritis in people (especially men) in their fifth decade or older [11, 12]. Despite this and the fact that hyperuricemia is recognized as the main risk factor for gout, the evidence that only a minority of patients with elevated serum uric acid levels develop gout suggests that other factors might be involved in determining crystal formation. In keeping with this, before the clinical expression of gout, a prolonged period of asymptomatic hyperuricemia often precedes the first attack of gouty arthritis, and an even longer period may be required for tophi to form. After their first attack, however, most untreated patients will experience a second episode within 2 years [11-13]. The progression from asymptomatic hyperuricemia to advanced gout is quite variable from person to person. In most people it takes many years to progress, if it does so at all. Nevertheless, despite the fact that significant heterogeneity has been described in the expression of gout, it has been proposed that a prototypic progression from asymptomatic hyperuricemia to chronic gouty arthritis could proceed through different stages of disease: 

### Stage 1:

Asymptomatic hyperuricemia. When serum urate concentration is greater than 6.8 mg/dL, urate crystals may begin to deposit. During this period of asymptomatic hyperuricemia, urate deposits may directly contribute to organ damage. This condition does not occur everytime, however, although there is an intense debate, it seems that there is no evidence that treatment is warranted for asymptomatic hyperuricemia.

### Stages 2 and 3:

Acute gout and intercritical periods. Developing urate deposits around joints, the local milieu or some trauma could activate the release of crystals into the joint space. In this case, a patient will suffer acute attacks of gout. These explosive pains are self-resolving but are likely to recur. In these intermittent phases, the intervals between attacks are termed “intercritical periods.” During these periods, crystal deposits may still be present at a low level in the fluid, and are certainly present in the periarticular and synovial tissue, providing a nidus for future attacks.

### Stage 4:

Advanced gout. If crystal deposits continue to accumulate, patients may develop chronically stiff and swollen joints. This is the advanced stage of gout and is relatively uncommon. Therapy could stop the progression and favour the avoidance of this advanced stage and its clinical expression. 

Besides the known arthicular and renal hyperuricemia-induced complications, high serum uric acid levels have been confirmed as a prognostic predictor of survival in heart failure. Furthermore, hyperuricemia associated with urate deposit has been identified as a risk factor for ischaemic heart disease, stroke, peripheral artheriopathy and renal failure. The risk persists even if other risk factors, such as metabolic syndrome components and hyperuricemic diuretics, are corrected [10-18]. 

Needless to say, hyperuricemia needs careful and regular monitoring. This approach is fundamental for a correct evaluation of the urate-lowering effectiveness of both nutritional and pharmacological therapy.

The Normative Aging Study is a longitudinal study that confirms the association between hyperuricemia and the development of urate deposition. A cohort of 2046 healthy volunteers have been monitored for 14.9 years. Urate deposition incidence was 0.1% in people with serum uric acid levels < 7.0 mg/dL; rising to 0.5% in people with uric acid levels from 7.0 to 8.9 mg/dL, and to 4.9% with uric acid levels higher than 9.0 mg/dL [10]. With urate levels of 9 mg/dl or higher, cumulative incidence of urate deposition reached 22% after five years. It is particularly interesting to point out that incidence rates were three times higher for hypertensive patients than for normotensive patients (p < 0.01). The strongest predictors of gout were age, body mass index, hypertension, cholesterol level and alcohol intake [10].

Acute gout is an acute inflammatory arthritis caused by intense inflammation secondary to chronic monosodium urate crystal deposition in joints. Gout episodes are spaced by asymptomatic/pauci-symptomatic periods, that can often be very long (years or decades). Nevertheless, gout clinical manifestations are multiple. Acute urate deposition most commonly begins with involvement of the first metatarsophalangeal joint (the so-called “podagra”), possibly evolving in a polyarticular arthritis, usually affecting ankle, knee and carpal joints. Extra-articular manifestations - particularly chronic nephropathy and uric acid nephrolithiasis - may occur both during acute episodes and asymptomatic periods. In any case, it is clinically relevant to point out how, before any symptoms occur, a constant and silent monosodium urate deposition has taken place during chronic hyperuricemia [1, 2, 9, 12]. This explains the importance of monitoring serum uric acid levels, especially in patients with metabolic risk factors. As a matter of fact, patients who after a first acute episode do not monitor or treat serum uric acid levels, usually experience a second acute episode within two years [11-13]. Progression from asymptomatic hyperuricemia to urate deposition may take years. Once urate deposition is clinically evident, it is fundamental to start a nutritional and pharmacological therapy (diet and hypouricemic drugs). In this phase, compliance is clearly very important, not only during the acute episodes but also during the asymptomatic periods spacing out the attacks.

## CHRONIC HYPERURICEMIA, URATE DEPOSITION AND CARDIOVASCULAR RISK 

**Fig. (2):**
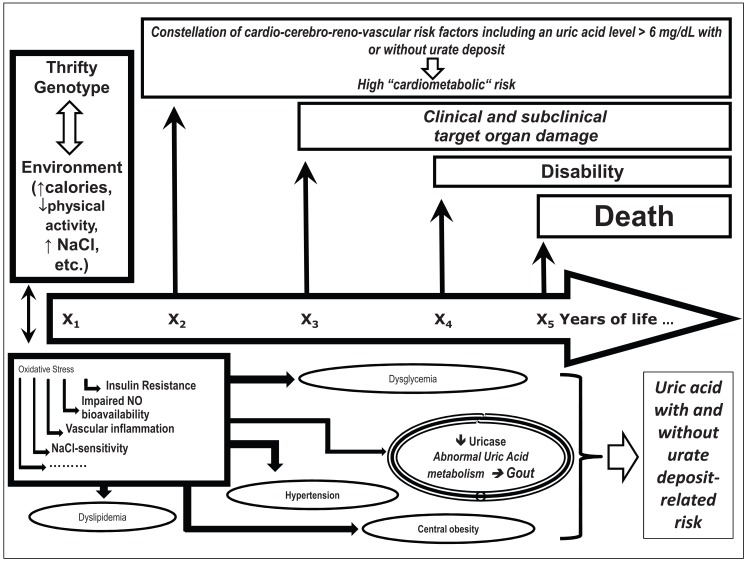
Negative interaction between thrifty genes and environment leads to the progressive appearance of a constellation of risk factors, including hyperuricemia with and without urate deposits. Claudio Ferri, personal drawing.

As already mentioned, hyperuricemia (both with and without urate deposition) and metabolic syndrome (defined as the coexistence of abnormal blood pressure, visceral obesity, dyslipidemia, and dysglicemia) are strongly associated (Fig. **2**). Concordantly, patients affected by chronic hyperuricemia and urate deposition manifest with an increased risk of developing coronary disease [14]. The Health Professionals Follow-up study showed that hyperuricemic and gouty arthritic patients had a higher mortality risk for cardiovascular diseases than those patients who had coronary disease [15]. Furthermore, hyperuricemic and gouty arthritic patients had a higher risk of developing metabolic syndrome than hyperuricemic and not gouty arthritic ones had [16]. Further studies showed an increased risk of myocardial infarction only in hyperuricemic patients with urate deposition (which means no increased risk in the ones without gout) after adjustments for renal function, metabolic syndrome, diuretics assumption and classic cardiovascular risks did not help decrease the risk [17, 18].

In addition to this, recent evidence has supported the concept that hyperuricemia itself can be a significant and independent cardiovascular risk factor, not only for cardiovascular and cerebrovascular diseases, but also for renal failure, hypertension and type 2 diabetes.

In agreement with this, hyperuricemia is common in people with higher cardiovascular risk: men, post-menopausal women (estrogens favor uric acid renal excretion), obesity, hypertension - especially the ones with organ damage - diabetes, dyslipidemia. According to this, we found increased - although not significantly - levels of serum uric acid in hypertensive patients with left ventricular hypertrophy compared to hypertensive patients without cardiac damage. Further, we also observed in hypertensive patients affected by metabolic syndrome a direct correlation between serum uric acid levels and body mass index, body weight, fasting insulin levels, and the index of insulin resistance HOMA-IR. 

Insulin resistance - in particular - might be responsible for an increased renal reabsorption of sodium and uric acid. This hypothesis is supported by the fact that 76% of chronic hyperuricemic with urate deposition patients are also affected by metabolic syndrome [19-22]. The strongest association occurs with visceral obesity, which is one of the components of the metabolic syndrome. In fact, visceral obesity is associated with hyperuricemia itself, with or without urate deposition [22, 23]. Takahashi *et al*. [23] observed a direct correlation between visceral fat quantity and serum uric acid levels (r=0.37, p<0.01), an inverse correlation with uric acid clearance (r=-0.34, p<0.05) and a direct correlation with the uric acid/creatinine ratio (r=0.65, p<0.0001). In agreement with this, Dessein *et al*. [21] studied the effect of weight loss and insulin-resistance on uric acid levels. They proved a 7.7±5.4 kg weight loss to be responsible for a reduction of monthly gout acute episodes (from 2.1±0.8 to 0.6±0.7 episodes per month, p=0.002). Moreover, serum uric acid normal levels were restored after a 58% weight loss in hyperuricemic patients.

Clinical and experimental studies have assessed the relationship between hyperuricemia and hypertension in the context of metabolic syndrome (regardless of the visceral obesity component this time). Hyperuricemia might play a double role as a risk factor for hypertension and as a pathological condition enhanced by hypertension itself. In Normative Aging Study [24] a total of 892 men developed hypertension over a mean of 21.5 years of follow-up of 2280 patients. Serum uric acid level independently predicted the development of hypertension in age-adjusted (relative risk [RR]: 1.10; 95% CI: 1.06 to 1.15: p<0.001) and glomerular filtration rate-adjusted (RR: 1.06; 95% CI: 1.01 to 1.12; p=0.03) models. These data assessed serum uric acid level as a valid marker of risk for the development of hypertension. The association is independent of elements of the metabolic syndrome, alcohol intake, and renal function. In agreement with this, recent studies have confirmed that hyperuricemia is also commonly associated with hypertension. It is present in 25% of untreated hypertensive subjects, in 50% of subjects taking diuretics, and in >75% of subjects with malignant hypertension [19].

Hyperuricemia has also been detected in borderline blood pressure patients [25], especially in the presence of micro-albuminuria [26]. Lee *et al*. [26] demonstrated that prehypertensive subjects (systolic blood pressure, 120 to 140 mmHg or diastolic blood pressure, 80 to 90 mmHg) with microalbuminuria had higher uric acid level than those with normoalbuminuria (men, 6.5±1.1 mg/dL versus 6.2±1.1 mg/dL; ***p***=0.017; women 4.8±0.9 mg/dL versus 4.4±0.9 mg/dL; ***p***=0.006). However, the difference in serum uric acid level according to the presence or absence of micro-albuminuria was not found in the normotensive group (systolic blood pressure, < 120 mmHg and diastolic blood pressure, < 80 mmHg). Multiple logistic regression models showed that, in the prehypertensive group, after adjustment for other cardiovascular risk factors, the highest uric acid quartile entailed > 2 times greater risk for microalbuminuria than the lowest quartile in both men (odds ratio, 2.12; 95% CI, 1.16 to 3.87) and women (odds ratio, 3.36; 95% CI, 1.17 to 9.69).

In the context of the association between hyperuricemia and cardiovascular risk factors, multiple studies have examined the specific correlation between hyperuricemia with and without urate deposition and cardiovascular events. In particular, uric acid values obtained on subjects of the original Framingham cohort predicted an increased risk of coronary heart disease and myocardial infarction in patients with high serum uric acid levels. The mean uric acid value for men was 5.0 mg/dl at the fourth examination and 5.7 mg/dl at examination 13 and was 3.9 mg/dl and 4.7 mg/dl, respectively, for women [27].

More recently, the preventive cardiology information system (PreCIS) database cohort study [28] observed that the mean uric acid levels were higher in patients previously diagnosed as having coronary artery disease than in the rest of the study patients (6.3 ± 1.7 mg/dl versus 5.9 ± 1.6 mg/dl; ***p*** < 0.001) and in the patients with diabetes mellitus than in the rest of the study patients (6.3 ± 1.8 mg/dl versus 6.0 ± 1.6 mg/dl; ***p***< 0.001). Uric acid levels were significantly higher in the subgroup of subjects who died compared with those who survived during the study period (7.1 ± 2.1 mg/dl versus 6.0 ± 1.6; ***p*** < 0.001). Kaplan-Meier analysis showed that although the risk of death increased gradually across the quartiles of uric acid values, only patients whose values were in quartile 4 (7.1-13.9 mg/dL) had significantly higher mortality rates as compared with those whose values were in quartile 1 (0.4-4.9 mg/dL), and the risk became evident at 3 years of follow-up. In particular, for every 1 mg/dl elevation in the serum uric acid level, the risk of death from all causes increased by 39% (95% CI 1.28-1.50, *P* < 0.001) [28]. 

After stepwise adjustment for age, sex, history of either diabetes mellitus, coronary artery disease, stroke, hypertension, smoking status, alcohol consumption, diuretic use, weight, BMI, systolic and diastolic blood pressure, fasting lipid levels, fasting glucose levels, and log-transformed GFR, the serum uric acid level remained a predictor of death from all causes (Hazard Ratio = 1.26 [95% CI 1.15-1.38], *p* < 0.001). The attributable risk per each standard deviation of uric acid for this model was 1.47 (95% CI 1.26-1.70, *p* < 0.001). Among the markers of metabolic syndrome, the serum uric acid level correlated with waist circumference (r = 0.35, ***p*** < 0.001), BMI (r = 0.27, ***p*** < 0.001), and triglyceride levels (r = 0.24, ***p*** < 0.001) and correlated inversely with HDL cholesterol (r = – 0.26, ***p*** < 0.001). Uric acid levels also correlated with serum creatinine levels (r = 0.31, ***p*** < 0.001) and homocysteine levels (r = 0.34, ***p*** < 0.001) [28].

Clearly, this study confirms serum uric acid levels as an indipendent predictor of mortality in populations at high risk of coronary artery disease. More recently, in a study by Ndrepepa *et al*. [29], 5.124 patients with acute coronary syndromes who underwent percutaneous coronary intervention (1.629 with acute ST-segment elevation myocardial infarction, 1.332 with acute non-ST-segment elevation myocardial infarction, and 2.163 with unstable angina) were divided into quartiles according to uric acid level as follows: quartile 1, 1.3 to <5.3 mg/dl; quartile 2, 5.3 to <6.3 mg/dl; quartile 3, 6.3 to <7.5 mg/dl; and quartile 4, 7.5 to 18.4 mg/dl. After 1 year follow-up they reported 80 deaths in quartile 1, 77 deaths in quartile 2, 72 deaths in quartile 3, and 221 deaths in quartile 4 of uric acid (Kaplan-Meier estimates of 1-year mortality 6.4%, 6.2%, 5.6%, and 17.4%, respectively; unadjusted hazard ratio 3.05, 95% confidence interval 2.54 to 3.67, p <0.001 for fourth vs first quartile of uric acid). Of note, after adjustment for traditional cardiovascular risk factors, renal function, and inflammatory status, the association between uric acid and mortality remained significant, with a 12% increase in the adjusted risk for 1-year mortality for every 1 mg/dl increase in the uric acid level [29]. 

## THERAPEUTIC PROSPECTIVES IN HYPERURICEMIA WITH URATE DEPOSITION

High uric acid blood levels can involve uric acid crystals articular and extra-articular deposition. This process is often silent, although it favors progressive joint destruction, renal failure and cardiovascular risk. As a consequence, uricemia control is fundamental, as well as uric acid levels monitoring over time. That is the reason why the European Guidelines [30] suggest serum uric acid levels ≤ 6 mg/dL in people having chronic hyperuricemia with urate deposition. Both hyperuricemia and tissue uric acid deposition are associated with cardiovascular risks. High serum uric acid levels speed up the atherosclerotic process and increase renal/cardiovascular morbidity and mortality rates. Nevertheless hyperuricemia, with or without urate deposition, is an independent risk factor for the above mentioned diseases. It follows obvious that uricemia must be considered in the context of a global cardiovascular evaluation.

Similarly, serum uric acid levels < 6 mg/dL have to be taken into consideration for a more complete reduction of the “cardiocerebral-vascular-renal risk” [1-3].

In this context, non-pharmacological therapy (diet poor in purine-rich food, sugars, alcohol and rich in vegetables and water intake) is critical. Despite their importance, dietary changes are scarcely prescribed. With regard to pharmacological therapy, it must be finalized to keep serum uric acid levels below 6.0 mg/dL. As in the previous case, pharmacological therapy is often underdosed or delayed [31].

As a fact, hypouricemic therapy is often limited only to acute episodes, due to a lack of compliance or to an underestimation of hyperuricemia toxic potential [1, 3, 12, 20, 31].

Hypouricemic therapy must be delayed until acute episodes are over. In fact, urate deposition is a chronic disease, which means that its correct treatment cannot be interrupted after the end of the acute episode.

Among hypouricemic drugs that are available in Italy, allopurinol has been the most used drug over the decades. Allopurinol is a purine analog; it is a structural isomer of hypoxanthine (a naturally occurring purine in the body) and is an inhibitor of the enzyme xanthine oxidase. Not always effective, it has been our main pharmacological option so far [1, 3, 12, 30, 31]. A new drug, febuxostat, has been available since december 2010. It is a purine-like selective inhibitor of the enzyme xanthine oxidase [31, 32]. Its therapeutic indication is chronic hyperuricemia with urate deposition and/or anamnestic report of tophi and/or gouty arthritis [31, 32].

**Fig. (3):**
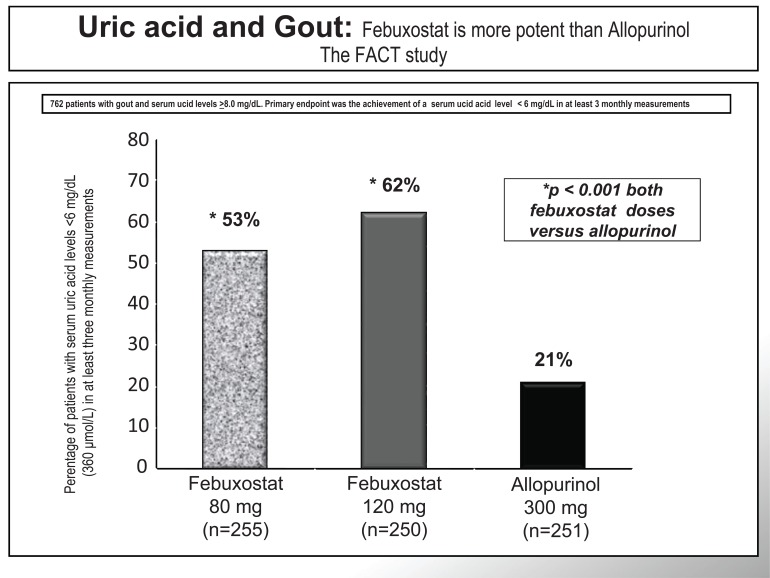
In the FACT study febuxostat reduced serum uric acid levels more than allopurinol in gouty patients with elevated serum uric acid levels. Primary endpoint was the achievement of a serum uric acid level < 6 mg/dL in at least 3 monthly measurements. Modified from ref 32.

**Fig. (4):**
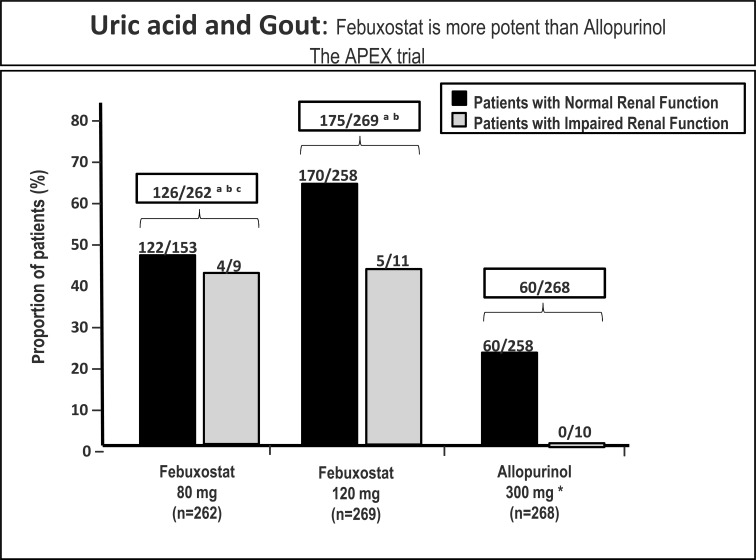
APEX trial: Patients with last 3 monthly serum urate levels <6.0 mg/dl in the intent-to-treat population. Treatment groups that were statistically significantly different for all patients were also statistically significantly different when the subset of patients with normal renal function was considered. * Ten patients received 100 mg and 258 subjects received 300 mg of allopurinol based on renal function. versus allopurinol in patients with impaired renal function; a = p < 0.05 versus allopurinol in all patients; b = p < 0.001 versus febuxostat 120 mg in all patients. c = p < 0.001 Modified from ref 33.

Febuxostat hypouricemic efficacy and tolerability have been widely assessed in recent clinical trials. Significantly higher percentages of subjects treated with febuxostat (80 mg and 120 mg) attained the primary end point of reaching and maintaining serum urate levels below 6.0 mg/dl (in agreement with European Guidelines), compared with those treated with allopurinol (300 mg/die) in the Febuxostat versus Allopurinol Controlled Trial (FACT) (Fig. **3**) [32]. It is important to mention that febuxostat excretion is both hepatic and renal, which made of febuxostat, in the APEX trial, a better choice for mild/moderate renal failure patients (Fig. **4**) [33]. For this reason febuxostat does not involve drug dose correction according to the renal impairment, as opposed to allopurinol. Moreover, the CONFIRMS trial established equivalent urate-lowering efficacy for febuxostat 40 mg daily and allopurinol 300/ 200 mg daily. At all levels of renal function studied, urate-lowering efficacy of febuxostat 80 mg daily was superior to that of febuxostat 40 mg or allopurinol 300/200 mg, and was comparably safe. In subjects with mildly or moderately impaired renal function, however, febuxostat 40 mg daily was significantly more effective in lowering serum uric acid levels than allopurinol. At the doses studied, safety of febuxostat and allopurinol, including cardiovascular safety, was comparable. Febuxostat, at 40 mg or 80 mg daily, offered a well-tolerated urate-lowering alternative to allopurinol, particularly for patients with mild or moderate renal impairment.

According to this, a recent review [35] summarizing all of the available information about the clinical use of febuxostat showed that cumulative clinical trial experience with this drug in both short- and long-term follow-up studies suggested it was well tolerated, with an adverse effect profile that was similar to those of placebo and allopurinol. Adverse effects were generally considered to be mild to moderate. Of note, this becames of major interest considering the ageing population is progressively growing and the incidence and prevalence of gout in the elderly is increasing. This appears related to improved lifespan leading to similar increases in age-related diseases (eg, cardiovascular and renal diseases) and their associated adverse effects of treatment (eg, diuretics and low-dose salicylates) which can increase the risk of gout. Several reviews have addressed the specific challenges of gout treatment in the elderly [36, 37]. In this regard, febuxostat might represent the new tool we need for the management of gout. Indeed, “*elderly onset gout*” differs from “classical” gout found in middle-aged men. The main differences are the more frequent subacute/chronic polyarticular onset with hand involvement, the unusual localization of tophi on ostheoarthritis nodes, an equal gender distribution (without male predominance), and the frequent association with drugs that decrease renal urate excretion (diuretics and low-dose aspirin) and/or with primitive renal impairment and the global multiorgan damage for age related diseases. Oxypurinol, which is the main metabolite of allopurinol and is responsible for most of its urate-lowering effects, is excreted predominantly by the kidneys and hence it has been recommended that the dosage of allopurinol could be reduced in patients with renal impairment. It has become increasingly obvious that alternative therapeutic options may have a significant impact on the future of successful gout management, especially in those patients with renal impairment or who are unresponsive or intolerant to allopurinol.

## CONCLUSION

Under a practical point of view, whatever the personal opinion a physician can have on the matter, it is our opinion that identification of possible causes, evaluation of renal function by appropriate formulas and non-pharmacological treatment should be taken into consideration in all patients with hyperuricemia, even in the absence of any specific uric acid-related disease. In conclusion, febuxostat data makes it a very interesting therapeutic option, that may lead to a new clinical management of hyperuricemia with urate deposition and their comorbidities. According to the most recent findings, hyperuricemia (with or without urate deposition) can be now considered as a component of the metabolic syndrome. It is an independent risk factor for renal and cardiovascular morbidity and mortality rates. However, clinical practice still needs further clinical trials finalized to assess urate-lowering efficacy in the much more global context of renal and cardiovascular prevention. This research might soon lead to a new possible therapeutic approach regarding hyperuricemia.

If extra-articular benefits of hyperuricemia treatment will be confirmed, hypouricemic therapy would then decrease the cardiovascular risk of treated patients as well as obviously improve their quality of life via a direct decrement in uric acid-related clinical events and complications. Safety and cost/benefit will also need further investigation in this specific field, especially regarding these new therapeutic strategies. However, this new therapeutic approach has already had a major role: it has brought back hyperuricemia and gout as a research element of interest. Chronic hyperuricemia with urate deposition has now achieved a whole new clinical meaning: it is an articular, renal and “cardiometabolic” disease.
